# Exceptional Performance of Flame-Retardant Polyurethane Foam: The Suppression Effect on Explosion Pressure and Flame Propagation of Methane-Air Premixed Gas

**DOI:** 10.3390/ma16247602

**Published:** 2023-12-11

**Authors:** Changhua Li, Guangyi Zhang, Bihe Yuan

**Affiliations:** School of Safety Science and Emergency Management, Wuhan University of Technology, Wuhan 430070, China; lich@whut.edu.cn (C.L.); zgy@whut.edu.cn (G.Z.)

**Keywords:** polyurethane foam, flame retardant, explosion suppression, explosion flame velocity, explosion overpressure

## Abstract

A self-built gas explosion testing platform was used to explore the quenching effect of flame-retardant polyurethane foam on a gas explosion. The effect of the foam’s filling position and length on the explosion suppression performance was explored. The results demonstrate that polyurethane foam exhibits an excellent flame-quenching performance, with a minimum of a 5 cm length of porous material being sufficient to completely quench the flame during propagation. Furthermore, the attenuation function of this porous material on the pressure wave is insignificantly affected by the change in ignition energy. Compared with the explosive state of the empty pipeline, the best suppression effect is obtained when the polyurethane foam is 20 cm in length with a filling position at 1.8 m, and the maximum explosion pressure and maximum rise rate are attenuated by 86.2% and 84.7%, respectively. This work has practical significance for the application of porous materials in explosion suppression and explosion-proof technologies in the chemical industrial processing and oil (gas) storage fields.

## 1. Introduction

With the development of society, the energy demand continues to increase. Natural gas, liquefied petroleum gas, and other flammable gases have been widely used in industrial processes and residential life. However, accidents caused by natural gas and petroleum gas leakages have also occurred frequently [1,2]. Flammable gases may have high explosion risks and hazards during their storage, transportation, and use. The ignition sources that may cause explosions are hot sources, electrical ignition, mechanically generated sparks, chemical ignition, and others, and these ignition sources with different energies are prone to trigger fire or explosion accidents [3,4]. Fire and explosion accidents have had serious consequences. Pipelines are an important gas transportation facility [5]. Effectiveness in suppressing the propagation of explosions and in attenuating explosion overpressure in pipelines is important, and it provides references for the application of porous materials in the field of process safety [6].

Scholars have made considerable progress in the field of gas explosion suppression technology. A large number of studies have focused on the use of inert gas [7,8,9,10], ultrafine water mist [11,12], and powder inhibitors [13,14,15] to suppress and mitigate gas explosions. Previous work explored the impact of various materials on the suppression of methane explosions. Yang et al. [16] explored the suppression mechanism of inertia isolation with inert gas for explosion flame propagation in pipelines. They proposed a solution of injecting the inert gas at multiple locations in the pipeline. This method inhibits ignition and flame propagation throughout the pipeline. Zhang et al. [17] analyzed the suppression effect of nitrogen (N_2_) and argon (Ar) on the explosion characteristics of dimethyl ether–air, and they noticed that N_2_ dilution is more effective than Ar in reducing the laminar burning velocity. NaCl is found to enhance the suppression effect of ultrafine water mist on methane explosions, reducing flame propagation velocity and explosion pressure [18]. Yang et al. [19] indicated that ultrafine water mist containing a methanotroph-inorganic salt had a suppression effect on a methane explosion. Different materials, including NH_4_H_2_PO_4_/red mud composite powder, Al(OH)_3_, and Mg(OH)_2_ powder, were tested for their abilities to suppress methane explosions [20,21,22].

In addition to the aforementioned explosion suppression technologies, porous media have been employed for explosion protection in fuel and oil storage tanks. Porous media, such as wire mesh [23], metal foam [24,25,26,27], and non-metal foam [28,29,30], have been investigated for explosion protection in fuel and oil storage tanks. To explore the quenching mechanism of porous media on a gaseous deflagration, researchers designed porous media with different geometric parameters and found that smaller pore sizes enhanced the flame quenching performance [31]. Zhuang et al. [32] studied the suppression of different porous materials on the explosion of flammable gases, and they proved that the thickness and pore size of the porous material had a marked influence on the explosion overpressure and intensity.

Porous materials are widely used in the field of explosion protection because they have many advantages, such as a three-dimensional structure, uniform pore size distribution, and high porosity, resulting in a high specific surface area. They have a suppressive impact on the chemical reactions of gas explosions. Traditional porous metal materials have several defects, such as easy oxidation, complicated installation, and replacement, and easy generation of metal chips. Polyurethane foam was introduced as a potential material for explosion protection due to its low density, easy installation, and controllable pore size. Herein, we report on the suppression effects of flame-retardant polyurethane foam on the explosion of flammable gas in pipelines.

## 2. Materials and Methods

### 2.1. Experimental Apparatus

**Figure 1:**
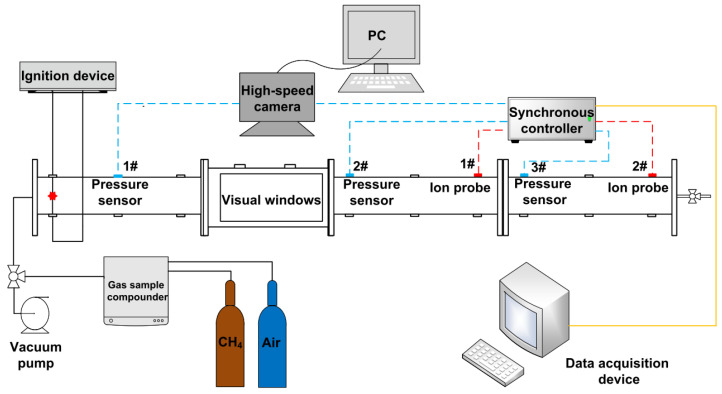
Explosion experimental platform.

The experimental setup (illustrated in Figure 1) includes a gas distribution system, data acquisition system, ignition device, and a horizontal pipeline with pressure sensors and ion probes. The high-speed camera uses an acquisition rate of 3000 frames/s. The ends of the pipe are closed. The ignition device adopts an electronic ignition method. The distance between the two electrodes is 3 mm, and the ignition duration is 40 ms. The ignition electrode is located 10 cm away from the front end of the pipe. The ignition energies are set as 100 mJ, 1 J, 10 J, and 20 J. The horizontal pipeline comprises three-section circular pipes with a diameter of 60 mm and a length of 2 m. The cross-section of the visual window is a rectangle with a side length of 60 mm and a length of 500 mm. The accuracy of the CYG508 miniature pressure sensor is 0.5% FS, and the maximum measuring range is 2 MPa. Figure 1 demonstrates the layout of the pressure sensor and ion probe on the pipeline. Three pressure sensors were placed at 1.2, 2.5, and 5.1 m away from the ignition source. Two ion probes were installed at 3.7 and 5.7 m away from the ignition source. The experimental material is flame-retardant polyurethane foam (FRPU-PN) with a pore size of 20 PPI and a diameter of 58 mm. It was provided by Dongguan Yangshui Industrial Co., Ltd. (Dongguan, China) The materials were fixed at 1.8 and 4.3 m away from the ignition source, and the material’s filling lengths are 5, 10, 15, and 20 cm.

### 2.2. Experimental Procedure

The concentration of methane in the air mixture is 9.5 vol%, according to its equivalent ratio. The first step of the experiment was to check the airtightness of the pipeline. After ensuring excellent airtightness, the second step was to use a vacuum pump to suck air from the pipeline, resulting in a vacuum state within the pipeline. The third step was to introduce the methane–air mixture to the sealed pipeline. Then, the explosion was triggered by the ignition device. The data acquisition system was used to record the flame propagation and explosion overpressure characteristic data under different conditions of filling length, ignition energy, and filling position.

### 2.3. Experimental Measurements

This work employed a STA6000 synchronous thermal analyzer (PerkinElmer, Waltham, MA, USA) for thermogravimetric analysis (TGA) to assess the thermal stability of the materials. The experimental parameters were set as follows: air atmosphere, heating rate of 20 °C/min, and a temperature range of 50–800 °C. The surface and cross-sectional morphology and structure of the samples were observed using a JSM-IT300 scanning electron microscope (SEM) (JEOL, Tokyo, Japan). Additionally, the limiting oxygen index (LOI) value of samples was measured using an AOI LOI apparatus (Motis Fire Technology Co., Ltd., Suzhou, China), according to ASTM D2863 standard [33].

## 3. Results and Discussion

### 3.1. Explosion Flame Propagation Characteristics

#### 3.1.1. Explosion Flame Propagation Characteristics of an Empty Pipeline

**Figure 2:**
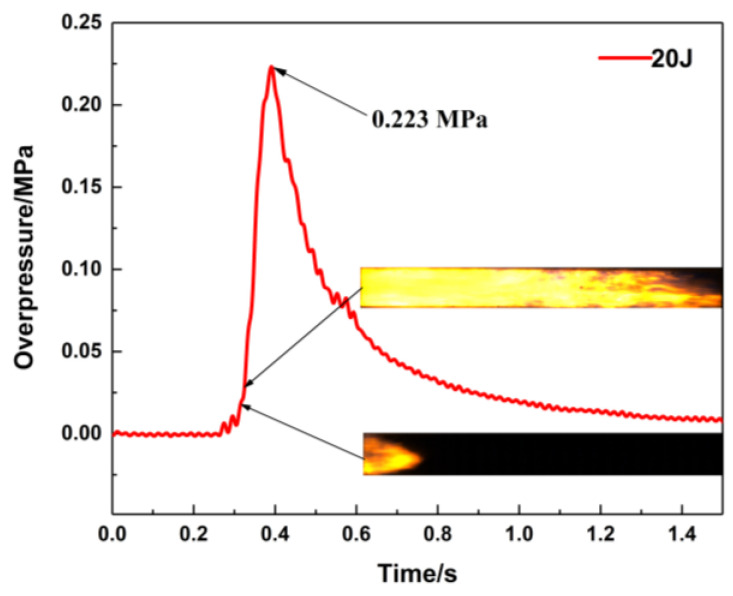
Pressure versus time curve (ignition energy: 20 J).

Methane–air mixtures are ignited under various conditions, and the effects on the flame propagation velocity and explosion overpressure are studied. Figure 2 shows the pressure curve obtained from the 2# pressure sensor and the corresponding flame propagation images of the methane–air premixed gas explosion in a closed pipeline in the absence of porous material. The arrival time of the pressure wave is earlier than that of the flame front, which indicates that the pressure wave velocity is faster than the flame front propagation velocity. The explosion overpressure does not reach its maximum value until the flame is quenched. According to the above analysis, it is proven that the flame propagation is closely related to the pressure wave propagation.

**Figure 3:**
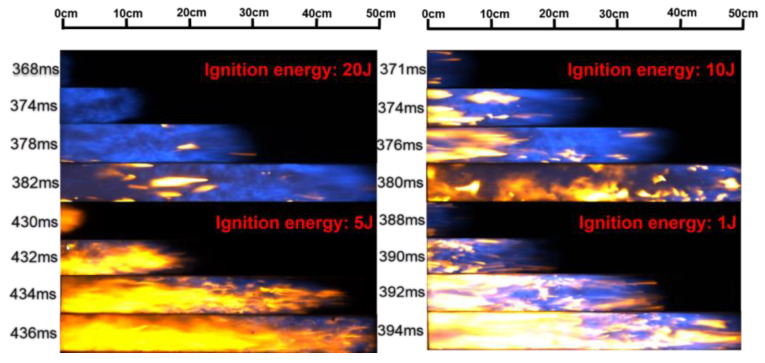
Flame propagation images under empty pipeline conditions.

The change in flame morphology is ascribed to the combined effects of the pressure wave and flame propagation velocity changes [34,35]. Figure 3 illustrates the flame propagation images in an empty pipeline with different ignition energies, i.e., 20 J, 10 J, 5 J, and 1 J. These flame structures exhibit some differences, but also have certain similarities when the flame passes through the visual window. When the flame front appears in the visual window, the flame structure is relatively regular, but there is no obvious contour structure. The reflection of turbulence causes the flame structure to be irregular and oscillated while propagating in the middle of the visual window, and the flame front is stretched into a wave or jagged shape [36]. Figure 3 points out that the flame propagation velocity is affected by the ignition energy.

**Figure 4:**
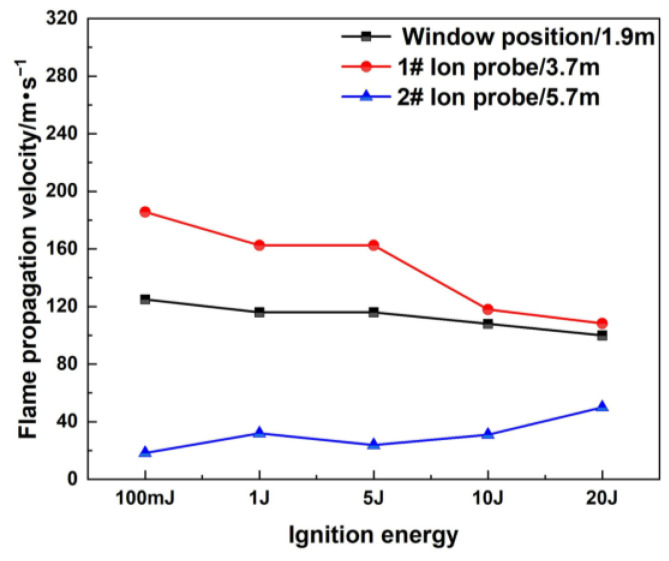
Flame propagation velocity under different ignition energies.

Figure 4 shows the flame propagation velocities under different positions and different ignition energies. The flame propagation velocity is the average value between the visual window and the ion probe, and between each ion probe. Under the same ignition conditions, the flame propagation velocity along the horizontal direction shows an initial increased tendency and then decreases [37,38]. Taking the flame propagation process of 100 mJ ignition energy as an example, the flame front moves slowly at the initial explosion stage. It takes 375 ms for the flame propagation to reach the position of the visible window, and the flame propagation velocity is 125 m/s at the visual window. As the explosion develops along the horizontal direction of the pipeline, the movement velocity of the flame front gradually accelerates, and the velocity is 185.7 m/s when the flame front reaches the position of the 1# ion probe. When the flame front approaches the closed end of the pipe, a reverse flow is generated and the propagation velocity decreases, and accordingly, the effect of heat loss increases, which further decreases the propagation velocity. The flame propagation velocity is 18.3 m/s at the 2# ion probe position. For different ignition energy conditions, it can be observed that the flame propagation velocity has a smaller range in the case of a higher ignition energy [39].

#### 3.1.2. Explosion Flame Propagation Characteristics after Filling Material

The polyurethane foam was evaluated for its flammability according to the limiting oxygen index (LOI). As depicted in Table 1, the polyurethane foam possesses an LOI value of 25.8%.

**Figure 5:**
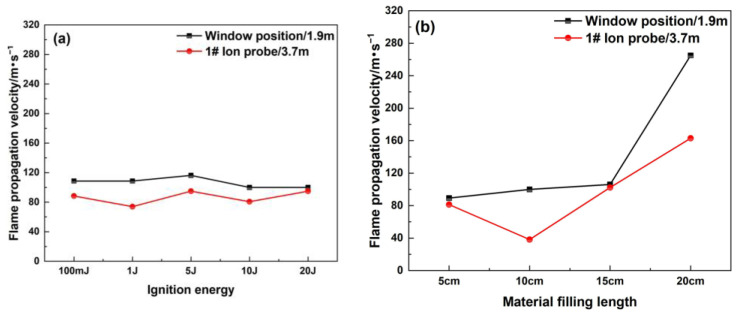
Flame propagation velocity after filling material: (**a**) different ignition energies (filling length: 20 cm); (**b**) different filling lengths (ignition energy: 20 J).

Flame-retardant polyurethane foam is examined for its ability to suppress explosions in pipelines under different conditions. Figure 5 shows the flame propagation velocity in the pipeline when the filling position is 4.3 m away from the ignition source, and the flame propagation velocity at the 1# ion probe becomes lower due to the blocking action of this material. The ion current curve of the 2# ion probe indicates that the value of the ion current does not change while filling the porous material, which proves that the porous material can completely quench the flame on one side of this material. The high specific surface area of the porous material and its three-dimensional framework can quickly absorb the heat carried by the flame entering the tortuous narrow channel, and quickly reduce its temperature until the flame is quenched. The above analysis shows that the changes in ignition energy and filling length have a great influence on the flame propagation velocity and flame structure, and they affect the explosion suppression performance of porous materials.

### 3.2. Explosion Overpressure Dynamics with Filling Material

#### 3.2.1. The Influence of Different Ignition Energies on the Explosion Overpressure

**Figure 6:**
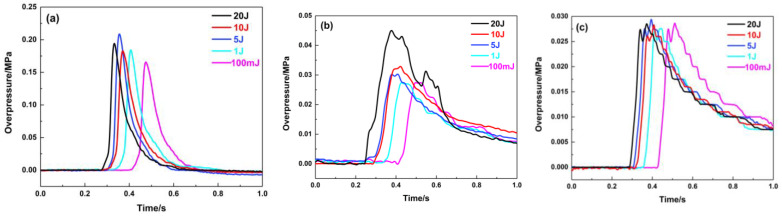
Explosion overpressure under different ignition energy conditions (filling position: 1.8 m): (**a**) 1# pressure sensor; (**b**) 2# pressure sensor; (**c**) 3# pressure sensor.

The maximum explosion overpressure is an important evaluation indicator for the hazards of a gas explosion. The positions of the porous material are located at 1.8 and 4.3 m away from the ignition source, and they have a significant suppression effect on the premixed gas explosion under different ignition energy conditions when the filling length is 20 cm. Figure 6 shows the pressure variation curves of the sensors at three different positions of the pipeline, where the porous material is at 1.8 m. The pressure curve of the 1# sensor shows that when the ignition energy is 100 mJ, the pressure begins to rise after 400 ms, gradually rises to the maximum value, and then gradually decreases. The whole curve is smooth and symmetrical. The explosion flame is quenched while passing through the porous material-filling area. The explosion fails to propagate along the pipeline. Thus, the pressure peak value decreases significantly while the pressure wave passes through the material. The pressure curve is tortuous and oscillates at the position of the 2# sensor. The overpressure continues to decay when the pressure wave propagates to the end of the pipeline. As the ignition energy becomes larger, there are more activated molecules per unit volume, and they can collide more effectively, so the mixed gas can be completely reacted in a shorter time, releasing much more energy. The pressure peak value also gradually increases, and the arrival time for the overpressure peak appears earlier. Compared with the explosion pressure curve in an empty pipeline, the maximum overpressure at the end of the pipeline is attenuated from 0.20 MPa to 0.03 MPa.

**Figure 7:**
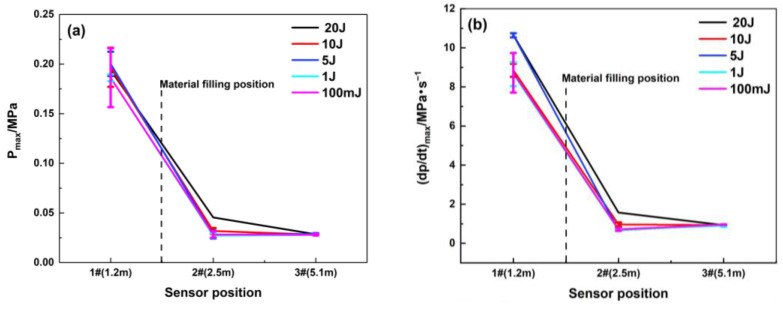
Maximum explosion overpressure and maximum rise rate of explosion overpressure under different ignition energy conditions (filling position: 1.8 m): (**a**) Pmax; (**b**) (dp/dt) max.

The maximum overpressure rise rate represents the maximum acceleration capability of a certain explosion field at a certain point. Figure 7 shows that the maximum overpressure rise rate is significantly suppressed, while the location of the porous material is filled at 1.8 m. Compared with the empty pipeline explosion, the suppression effect of this porous material is obvious, and the maximum rise rate decays by 84.7%. The gradient of ignition energy set in the tests is relatively small; thus, the difference between the maximum explosion overpressure and maximum rise rate for each ignition energy is relatively non-significant, and the change law is not obvious.

**Figure 8:**
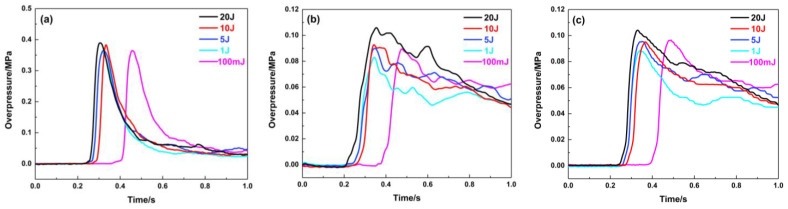
Explosion overpressure under different ignition energy conditions (filling position: 4.3 m): (**a**) 1# pressure sensor; (**b**) 2# pressure sensor; (**c**) 3# pressure sensor.

Figure 8 and Table 2 indicate that the pressure suppression effect of the material filling at the 4.3 m position is obvious. Compared with the working condition at 1.8 m, the suppression effect is insufficient. When the distance of porous material from the ignition source is 1.8 m, the material can restrain the maximum overpressure rise rate to 2 MPa/s, and the maximum explosion pressure value is below 0.05 MPa. The maximum overpressure rise rate at 4 m is 6 MPa/s, and the maximum explosion overpressure value is below 0.125 MPa. Under the same filling length and ignition energy conditions, when the material is filled at 4.3 m, the characteristic values of explosive overpressure are higher than those of the material filled at 1.8 m. The above analysis confirms that the filling position of the porous material significantly influences the suppression effect on the explosion overpressure. While the ignition energy gradually increases, the attenuation effect of the porous material on the maximum explosion pressure and maximum rise rate at the same position is still significant.

#### 3.2.2. The Influence of Different Filling Lengths on Explosive Overpressure

**Figure 9:**
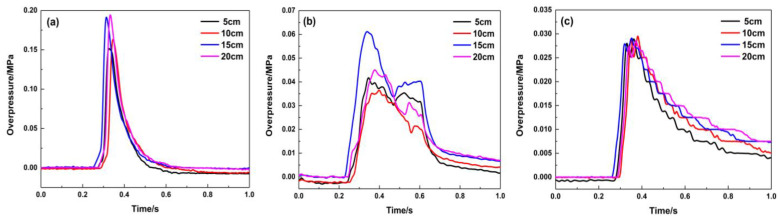
Explosion overpressure under different filling lengths (filling position: 1.8 m): (**a**) 1# pressure sensor; (**b**) 2# pressure sensor; (**c**) 3# pressure sensor.

**Figure 10:**
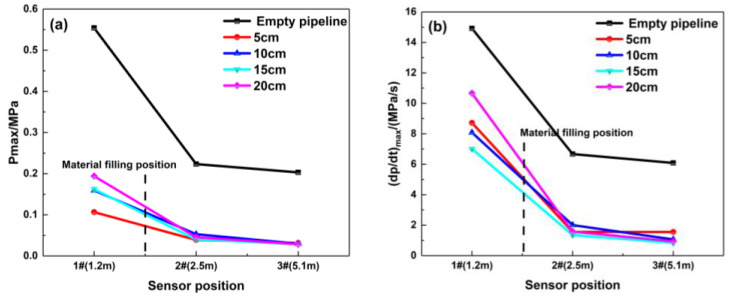
Maximum explosion overpressure and maximum rise rate of explosion overpressure under different filling lengths (filling position: 1.8 m): (**a**) Pmax; (**b**) (dp/dt) max.

The filling length of porous material has a significant impact on the pressure in the middle and start of pipeline, and changing the length has an unmarked effect on the pressure at the end of the pipeline. As shown in Figure 9, when the material is filled at 1.8 m, the explosion in the pipeline is not developed due to the material’s restrictions on the explosion. The explosion overpressure curve in the front of the pipeline is very low, and it is smooth, which indicates that the filling material has little influence on the pressure wave in this area. Due to the high flow resistance of porous materials and the “consumption” of pressure waves by their pores, the maximum explosion overpressure decreases to below 0.06 MPa when the pressure waves pass through the material-filling area, and it shows that the filling length of this material has a significant impact on the pressure in the middle of the pipeline. While the pressure wave reaches the end of the pipeline, the effect of the filling lengths on the pressure peak becomes smaller. Figure 10 indicates that the porous material has great suppression performance and can suppress the pressure wave of the premixed gas explosion from further propagation. When the material filling length is 20 cm, the attenuation effect of the porous medium on the pressure wave is the most obvious at the end of the pipeline, and the maximum explosion pressure and maximum rise rate are attenuated by 86.2% and 84.7%, respectively.

**Figure 11:**
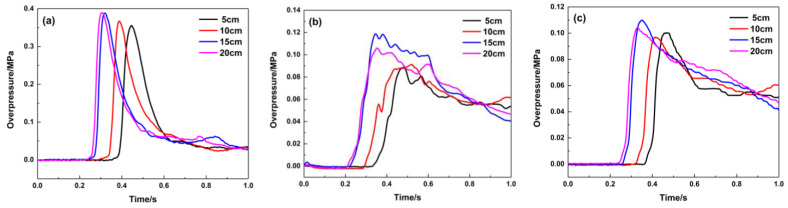
Explosion overpressure under different filling lengths (filling position: 4.3 m): (**a**) 1# pressure sensor; (**b**) 2# pressure sensor; (**c**) 3# pressure sensor.

Figure 11 graphically describes the pressure curve while the material is filled at 4.3 m. Its suppression effect on the explosion overpressure is similar to that of the filled material at 1.8 m. Compared with the pressure curve in Figure 9, the maximum explosion overpressure values at the middle and end of the pipeline are approximately 0.1 MPa, indicating that its suppression effect is weaker than that at 1.8 m. Table 3 shows that the explosion overpressure and its rising rate are suppressed. While this material is filled at 4.3 m, the porous medium has the greatest impact on the pressure wave in the middle of the pipeline, and the maximum explosion pressure and maximum rise rate are attenuated by 51.5% and 65.6%, respectively.

### 3.3. The Suppression Mechanism of Porous Materials in Gas Explosions

**Figure 12:**
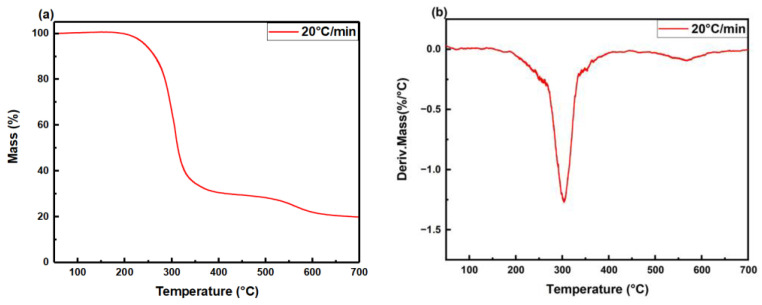
(**a**) TGA and (**b**) DTG curves of polyurethane foam in air atmosphere.

To further explore the thermal stability of the polyurethane foam, a thermogravimetric test was conducted on the sample in an air atmosphere with a heating rate of 20 °C/min. Figure 12 illustrates the thermogravimetric analysis (TGA) and differential thermogravimetric (DTG) curves of the polyurethane foam. The relative thermal stability of the sample is evaluated based on typical decomposition temperatures. According to the analysis of the TGA curve (Figure 12a), the polyurethane decomposition process can be divided into two stages. During the initial decomposition stage (240–400 °C), the polybasic alcohols in the sample undergo oxidation, resulting in the formation of CO, CO_2_, and H_2_O, which leads to a substantial mass loss of approximately 70%. In the second thermal decomposition stage (510–620 °C), the remaining chain segments within the sample continue decomposing, and there is a decline in the mass loss rate, where the polyurethane foam loses about only 10 wt% [40,41]. This experiment shows that the polyurethane foam in this work has considerable thermal stability.

**Figure 13:**
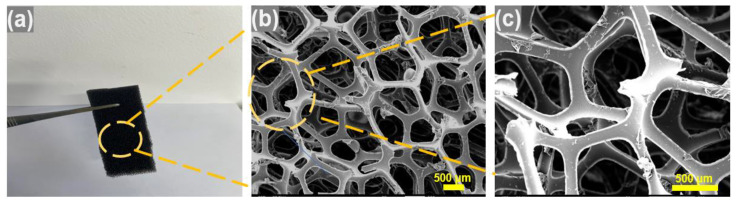
(**a**) Digital photos and (**b**,**c**) SEM images of polyurethane foam.

The study suggests that polyurethane foam’s three-dimensional structure and high porosity contribute to the flame quenching and attenuation of pressure waves in gas explosions. The experimental results also prove that the polyurethane foam successfully extinguishes the flame in the pipeline. Figure 13 depicts digital photographs and scanning electron microscopy (SEM) images of the polyurethane foam. The SEM image of the polyurethane foam (Figure 13b) demonstrates its three-dimensional pore structure, uniformly distributed pores, high porosity, and high specific surface area. These structural characteristics grant the material significant advantages in flame retardancy and explosion suppression, playing a crucial role in inhibiting methane–air premixed gas explosions. Its three-dimensional network structure, upon encountering an explosion flame, forces the flame to fragment into smaller flames. The high surface area of this porous material ensures that the explosion flame can make sufficient contact with the material when it propagates into the pore spaces, which enables the explosion flame to transfer more heat to the pore structure [42,43] and reduce the temperature of the flame. The combustion reaction cannot propagate to the unreacted area, and the flame is extinguished [44,45,46].

In addition to flame quenching, porous media also have a significant attenuation effect on pressure waves. The regularly distributed pore structure effectively divides, reflects, and scatters pressure waves, and the internal network pore skeleton undergoes elastic deformation to consume the energy of the pressure wave. It leads to continuously consumed pressure-wave energy [47,48] and reduces the maximum explosive overpressure at the back of the material.

## 4. Conclusions

Explosion suppression experiments using polyurethane foam were conducted in a closed pipeline with methane–air premixed gas. Experimental results revealed the effective quenching of flame propagation by the pore structure of polyurethane foam when the explosion flame passes through small pores. The study investigated the impact of different ignition energies and foam filling methods on flame propagation, explosion overpressure, and pressure rise rate. With a filling length of 20 cm and a filling position at 1.8 m, the polyurethane foam demonstrated significant suppression effects, reducing the maximum explosion pressure and maximum rise rate by 86.2% and 84.7%, respectively. The flame-retardant polyurethane foam, characterized by low density and uniform pores, holds promise for explosion-proof applications in oil and gas storage tanks at petrol stations.

This research aims to mitigate the damage caused by explosions of flammable gases and to prevent the escalation of fire and explosion incidents, holding profound significance for the prevention and control of fire and explosion disasters related to flammable gases. Due to the complexity of the explosion suppression mechanism of porous materials and limited research depth, there are still several shortcomings in this research work, necessitating further research processes:

(1) We need to explore the evolution behavior of the flow field during the process of inhibiting premixed gas explosions using porous materials, in order to conduct a more in-depth study of the inhibition mechanism of porous polyurethane composites.
(2) We need to investigate the inhibitory effects of a porous polyurethane composite on explosions of different hazardous materials, such as hydrogen gas and oil–gas mixtures. Simultaneously, by adding various flame retardants, we can broaden the applicable scenarios of porous polyurethane composites to meet a wider range of functional requirements.

## Figures and Tables

**Table 1:** LOI tests for the polyurethane foam.

Sample	LOI (%)
polyurethane foam	25.8%

**Table 2:** Maximum explosion overpressure and maximum rise rate of explosion overpressure under different ignition energy conditions (filling position: 4.3 m).

Ignition Energy	Maximum Explosion Overpressure (MPa)			Maximum Rise Rate of Explosion Overpressure (MPa/s)		
	1#	2#	3#	1#	2#	3#
20 J	0.3787	0.1089	0.1029	16.0195	3.2988	3.1626
10 J	0.3735	0.0944	0.0909	16.8995	3.2410	2.7247
5 J	0.3804	0.1013	0.1009	16.4790	3.1139	2.9709
1 J	0.3715	0.0925	0.0908	16.1989	3.4488	2.9447
100 mJ	0.3825	0.0944	0.0955	17.2573	4.3074	2.8957

**Table 3:** Maximum explosion overpressure and maximum rise rate of explosion overpressure under different filling lengths (filling position: 4.3 m).

Filling Length	Maximum Explosion Overpressure (MPa)			Maximum Rise Rate of Explosion Overpressure (MPa/s)		
	1#	2#	3#	1#	2#	3#
Empty pipeline	0.5541	0.2232	0.2030	14.9187	6.6706	6.0854
5 cm	0.3453	0.1005	0.0984	13.3321	2.1285	3.4076
10 cm	0.3481	0.1025	0.0943	14.1356	2.3228	2.9677
15 cm	0.3788	0.1117	0.1012	16.3142	3.2885	3.4068
20 cm	0.3787	0.1089	0.1029	16.0195	2.2988	3.1626

## Data Availability

The data presented in this study are available upon request from the corresponding author.
